# Norwegian kidney biopsy biobank (NorKiBB): organization, baseline characteristics, and generalizability of a low-cost national biobank

**DOI:** 10.1186/s12882-025-04007-4

**Published:** 2025-02-13

**Authors:** Marius Altern Øvrehus, Knut Asbjørn Rise Langlo, Sabine Leh, Øystein Eikrem, Solfrid Romundstad, Håvard Aksnes, Ingjerd Wangensteen Manner, Christian Aalborg, Marit D. Solbu, Lasse G. Gøransson, Hans-Peter Marti, Michael G. Shlipak, Joachim H. Ix, Stein I. Hallan

**Affiliations:** 1 Department of Clinical and Molecular Medicine, Faculty of Medicine and Health Sciences, Norwegian University of Science and Technology, Trondheim, Norway; 2 Department of Nephrology, St. Olavs Hospital, Trondheim University Hospital, P.b. 3250, Trondheim, Torgarden, NO 7006 Norway; 3 Department of Pathology, Haukeland University Hospital, Bergen, Norway; 4 Department of Nephrology, Haukeland University Hospital, Bergen, Norway; 5 Department of Internal Medicine, Nephrology Unit, Levanger Hospital, Levanger, Norway; 6 Department of Internal Medicine, Nephrology Unit, Lillehammer Hospital, Lillehammer, Norway; 7 Department of Nephrology, Oslo University Hospital, Ullevål, Oslo Norway; 8 Department of Nephrology, Akershus Oslo University Hospital, Akershus Universitetssykehus, Lørenskog, Norway; 9 Department of Nephrology, University Hospital of North Norway, Tromsø, Norway; 10 Department of Nephrology, Stavanger University Hospital, Stavanger, Norway; 11 Department of Medicine, San Francisco Veterans Affairs Healthcare System, Kidney Health Research Collaborative of San Francisco, University of California, San Francisco, CA USA; 12 Division of Nephrology-Hypertension, Department of Medicine, San Diego, and Veterans Affairs San Diego Healthcare System, University of California, San Diego, CA USA

**Keywords:** Biobank, Chronic kidney disease, Epidemiology, Glomerulonephritis, Kidney biopsy

## Abstract

**Background:**

Biobanks that hold blood, urine and kidney tissue are key for translational nephrology research but are few and have limited availability. We describe the organization, baseline characteristics, and generalizability of a low-cost national biobank.

**Materials and methods:**

Eight Norwegian hospitals participated in this multi-center, prospective cohort study and biobank initiative. Patients referred for routine clinical native kidney biopsies were eligible for inclusion, starting September 2020. Extensive information on medical history and risk factors were collected into an encrypted on-line database by the treating nephrologist. A comprehensive standardized panel of blood and urine tests were analyzed in the clinical routine and registered along with the full histology report. Extra urine and blood samples were collected, aliquoted and prepared locally within two hours, frozen at -80 C, and later sent to a central government-funded biorepository together with remaining kidney biopsy material.

**Results:**

By September 2023, a total of 633 patients were included out of 1050 eligible patients. Mean age was 52.6 years (SD 18.7), 384 (61%) were men, and participants displayed a wide spectrum of kidney disease with mean estimated glomerular filtration rate (eGFR) 53 mL/min/1.73m^2^. The most frequent biopsy indications were progressive chronic kidney disease (CKD) of unknown cause, acute kidney disease, and isolated hematuria/proteinuria. The most frequent diagnoses were IgA nephropathy (21%), arterionephrosclerosis (13%), and diabetes nephropathy (9%). Biopsy indications and diagnoses were similar to the spectrum typically seen in Norway and other western countries, and similar population level kidney health measures were demonstrated for Norway, United Kingdom, and USA.

**Discussion:**

We demonstrate the feasibility of establishing a large national kidney biopsy biobank across a variety of clinical and histopathologic diagnoses. Blood and urine were stored, accompanied by kidney tissue, at a moderate cost due to a combination of a dedicated nephrology workforce, routine clinical care, and established biobank facilities.

**Supplementary Information:**

The online version contains supplementary material available at 10.1186/s12882-025-04007-4.

## Background

Chronic kidney disease (CKD) is highly prevalent in adults worldwide and is associated with poor individual and public health. CKD can progress to kidney failure needing dialysis or transplantation and carries a high risk of cardiovascular complications [1]. Kidney biopsies can give insights to unique etiologies, and key characteristics like the severity of interstitial fibrosis can provide insights to severity of disease and prognosis. However, biopsies are invasive and rarely done. Because current clinical biomarkers of kidney health (estimated glomerular filtration rate (eGFR) and albuminuria) do not capture or distinguish these biopsy features, a major limitation in our ability to prevent and treat CKD has been the absence of non-invasive diagnostic tests. Without tests that distinguish between different underlying disease processes, treatments most often are not specific to the individual’s disease. While multiple new non-invasive kidney biomarkers have been identified, their relation to different kidney disease processes and histopathologic features remain unknown. Novel diagnostic strategies to distinguish the causes of CKD have been hindered by the lack of access to human studies with tissue samples including kidney biopsies [2–5].

There are few existing kidney biopsy biobanks, and those that exist have important limitations. Often such biobanks are limited to small sample sizes and single centers, which almost certainly will reflect local biopsy referral patterns and may limit statistical power. Only a minority of existing biopsy biobanks have consistently and concurrently obtained blood and urine specimens in order to evaluate relationships of biomarkers with histopathologic features [6–8].

In Norway, all kidney biopsies are registered and stored locally, evaluated by nephro-pathologists regionally, and a digitalized whole biopsy slide and the histology report is sent to a single academic center in Bergen, Norway, to form part of the Norwegian Renal Registry, which serves as a national quality registry [9]. This provides an opportunity to examine all kidney biopsies across the entire country, and the ability to leverage collaborations to collect blood and urine before planned clinical biopsies. Extensive clinical phenotyping and long-term follow-up is achievable from all Norwegian participants by linkage to their electronic patient records. Patients may also be linked to existing national quality registries through their unique national eleven-digit personal identification number.

We aimed to establish a Norwegian kidney biobank with urine, blood, and kidney tissue from patients referred for routine clinical native kidney biopsies. Here we describe the establishment and the structure of the biobank, baseline characteristics of patients included during the initial 3-year period, and we conclude with a discussion of the feasibility and generalizability of this biobank.

## Methods

### Objectives

The aim of the Norwegian Kidney Biopsy Biobank (NorKiBB) is to improve knowledge and outcomes for CKD patients by providing researchers access to high quality bio-specimens and associated clinical data concurrent with kidney biopsy data. To this end, we wanted to establish a population-based cohort to provide generalizable results. This biobank initiative aims to improve the collaboration among clinical, pathological, epidemiological, molecular, and genetic research environments to generate new knowledge generalizable to the clinical kidney communities.

### CKD healthcare in Norway

All healthcare in Norway is organized and funded by the national government, and all levels of care are free for all citizens. General practitioners serve as gatekeepers and decide whether a referral to a nephrologist or hospital admittance is needed. Kidney biopsies are performed by radiologists or nephrologists at 20 different hospitals across Norway, and further histopathological examination is conducted at one of the four university hospitals that have a dedicated nephro-pathology service. Currently, the kidney biopsy frequency is 98 per million population per year [9]. The incidence of kidney failure necessitating renal replacement therapy (RRT, i.e. dialysis or transplantation) is 98 per million per year, and 10% of RRT patients receive a preemptive kidney transplant [10].

### Recruitment of patients

**Fig. 1:**
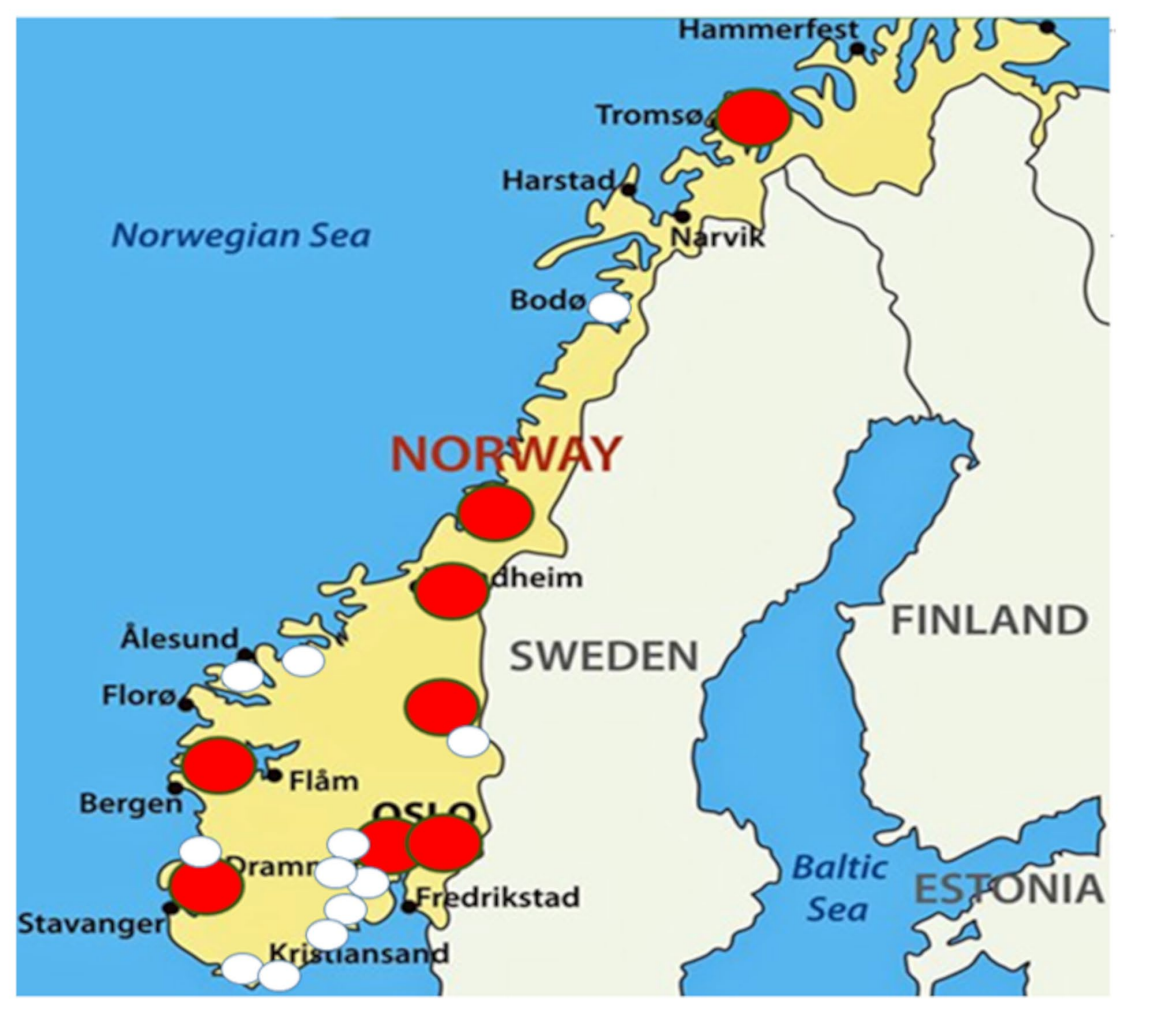
A map of Norway, with the participating centers (red dots) and the non-participating centers (white dots). Source and copyright: The authors

All hospitals that perform kidney biopsies in Norway were invited to participate (Fig. 1). All adult patients referred for a routine kidney biopsy by their treating nephrologist on clinical indication were eligible for inclusion. Patients were excluded if they were not able or willing to give a written informed consent. Inclusion started September 2020. Recruitment is still ongoing.

### Collection and preparation of bioresources

All participants undergoing native kidney biopsy donated blood and urine at the outpatient clinic or the inpatient ward during the 48 h prior to the biopsy procedure. All centers used the same standard operating procedure (SOP) for collection, preparation, aliquoting, and storage of urine, blood, and kidney tissue. In addition to standard biochemical tests needed for full evaluation of kidney diseases and biopsy preparation, extra blood and urine were obtained for long-term storage in the biobank. Blood was drawn for 2 × 5 mL Vacutainer SST II serum tubes, then centrifuged at 2200 G for 10 min after 30 min in room temperature, aliquoted into 0.5mL tubes and frozen at -80 °C. Blood was also drawn for 3 × 6 mL Vacutainer EDTA tubes, immediately centrifuged at 2200 G for 10 min at 4 °C, plasma was aliquoted into 0.5mL tubes, and the remaining plasma and buffy coat (leucocytes and thrombocytes) was transferred to 0.5mL tubes. All tubes were immediately frozen at -80 °C. Sixty to 100 mL of mid-stream second urine of the day was collected in a 120 mL plastic cup and transferred to 6 × 10 mL vacutainer tubes. Two 4.5 mL aliquots of raw urine were frozen at -80 °C without centrifugation or additives. The remaining urine was centrifuged at 1000 G for 12 min at 4 °C, and protease inhibitor (Protease Inhibitor Cocktail P1860, Sigma) was added to 4 × 4.5 mL aliquots. Another six 4.5 mL aliquots were frozen without protease inhibitor. We added RNA-later (R0901, Sigma) to the remaining cell pellets and transferred the solution to three 0.5 mL tubes. Kidney biopsy samples were handled by the standard protocols of each of the four nephropathology centers, including ordinary preparation for standard histological staining for light microscopy, immunofluorescence or immunohistochemistry, and electron microscopy. All slides were described histopathologically by two of the nephro-pathologists at the regional pathology center. Subsequently, four unstained slides were prepared from the remaining material and stored at -80 C.

### Storage of biological samples

Blood and urine aliquots were stored locally and later sent to Biobank1 in Trondheim in batches of 20 patients. The samples were packed in Styrofoam boxes filled with dry ice and sent express with a commercial courier to the biobank with transportation time less than 24 h. Biobank1 is a joint project between the Norwegian University of Science and Technology (NTNU) and the Mid-Norway Regional Health Authority to store data and biological samples for research purposes in Central Norway. Biobank1 organized de-identification of data, barcoding samples, storage and tracking of samples and related information. Samples were stored according to best practice, at -80 °C with temperature logging and alarm system. Kidney biopsies were also scanned and deposited digitally in the Norwegian Renal Registry.

### Data security and collection of medical history and risk factor data

We used a web-based system for registration of medical data (eForsk, Helse Midt-Norge IT, Norway). This is a two-way encryption solution with two-factor authentication enabling safe transfer of sensitive patient information via the closed Norwegian Health Network. In this way, patient identifiable information can be entered locally by the treating nephrologists who have the most comprehensive medical information of the patient and transferred directly to the biobank without the need for a local encryption key. Standardized questions on medical history, indication for biopsy, risk factors, medications, diagnostic tests (antineutrophil cytoplasmic antibodies ANCA, antinuclear antibodies ANA, complement, and other serological testing), procedure complications, and other queries were answered by the local nephrologist using a combination of pull-down menus and free text sections. A complete list of variables is available in the Supplementary Table 1.

### Biochemical tests used for the baseline kidney evaluation

To fully characterize all patients at the time of the kidney biopsy, a standard test panel was analyzed locally. This included a complete blood cell count, bleeding risk evaluation, electrolytes, kidney function, analytes for glucose and lipid metabolism, and others. Urine was tested with a dipstick, and electrolytes, albumin, creatinine, and osmolality were quantified. See Supplementary Table 1 for complete list of the 42 analytes measured. All measurements were done in the clinical laboratories of the participating hospitals.

### Kidney biopsy procedure and histological examination

The most widespread biopsy procedure in Norway is for patients to have two kidney core biopsies taken with a 16G needle. The biopsies are sent to the regional nephro-pathology laboratory, where routine examinations are performed: standard light-microscopy staining (hematoxylin-eosin HE, Periodic acid-Schiff PAS, silver, Congo red), immunofluorescence or immunohistochemistry (IgA, IgG, IgM, C3, C4, C1q, kappa and lambda free light chains), and electron microscopy (in > 90% of the cases). Results are then typically examined by two nephro-pathologists. The clinical reads from the nephro-pathologists were used as the biopsy description in our biobank.

Data from all Norwegian kidney biopsies, i.e., both those included in the NorKiBB and those not participating, are sent to the Norwegian Renal Registry. This is a national quality registry for patients with CKD stage 5, renal replacement therapy, or kidney biopsies. The entries include the full histology report, a selected sub-set of clinical and laboratory data, a HE stained digitalized whole slide image, but no biospecimens.

### Ethics

The NorKiBB was approved by the Regional Committee for Medical Research Ethics Central Norway, as well as by the local data protection boards/data access committees in all of the participating hospitals: the Data Protection Officer at St. Olavs Hospital, Trondheim University Hospital, Trondheim; the Regional Committee for Medical Research Ethics Western Norway for Haukeland University Hospital, Bergen; the Data Access Committee at Levanger Hospital, Levanger; the Data Protection Officer at Lillehammer Hospital, Lillehammer; the Data Protection Officer at Oslo University Hospital, Ullevål, Oslo; the Data Protection Office at Akershus Oslo University Hospital, Lørenskog; the Data Protection Official at the University Hospital of North Norway, Tromsø; and The Research Department at Stavanger University Hospital, Stavanger. All patients gave their written, informed consent after having received oral and written information about the project, possible disadvantages, and benefits in accordance with the General Data Protection Regulation (GDPR). A copy of the consent form is available in the Supplementary section. All experiments were performed in accordance with the Norwegian Act on medical and health research, and the Personal Data Act. The experiments were conducted in accordance with the Declaration of Helsinki.

### Funding and costs

The NorKiBB was established as part of the project “Kidney Tubular Damage and Dysfunction Identify a Novel Axis of Chronic Kidney Disease”. This is a joint US - Norwegian project funded by the U.S. National Institute of Health and the Norwegian Research Council. The project aims to assess the relationships of a wide range of blood and urine tubulointerstitial biomarkers with histopathological manifestations of kidney health. A Norwegian national biobank with data and samples from patients undergoing clinical kidney biopsy was initiated with the aim to collect sufficient material to enable further research projects beyond the Kidney Tubular Damage project. The biobank was named NorKiBB. Recruitment of patients, registration of data, analyses for standard CKD evaluation, and histopathology were considered part of the ordinary hospital practice and therefore not directly reimbursed. The laboratories were reimbursed for extra materials and personnel needed for preparation, registration, local storage, and transportation of biomaterial to BioBank1. Biobank1 also charges a rather small annual amount for storage of samples, so the total costs for establishing and including this first wave of patients were rather low.

### External resources of relevance for the realization of the biobank

Linkage to external resources is quick and accurate using the unique eleven-digit identification number given to all Norwegian citizens at birth or at immigration. Norway has a long tradition for medical registries, and mandatory reporting to multiple central registries can give highly relevant information for kidney researchers (e.g., medical birth registry, cause of death registry, medical drug prescriptions registry, cancer registry, and others). In addition to the kidney disease registry mentioned above, there are similar registries for myocardial infarction, heart failure, stroke, peripheral vascular disease, diabetes, bariatric surgery, vascular surgery, vasculitis, and others.

The public specialist health care system in Norway is mainly funded by the Norwegian federal government and organized in regional health trusts. Private nephrology clinics are almost non-existing. For CKD patients, this means that their general practitioner, the nephrologist at the outpatient clinic, and all laboratories and hospitals are part of the same organization. This enables unique possibilities for cooperation for both clinical practice and research.

### Governance

The NorKiBB is a very small organization with no personnel directly employed at the biobank. The Steering Committee is composed of active kidney researchers from the five Norwegian university clinics in Trondheim, Tromsø, Bergen, and Oslo (see Acknowledgements). They provide general oversight of the biobank activities, review and adjudicate incoming research application requests, and discuss future targets and funding opportunities. Day to day activities are managed by the Kidney Research Group at NTNU/St. Olavs Hospital and by personnel at Biobank1.

## Results

**Fig. 2:**
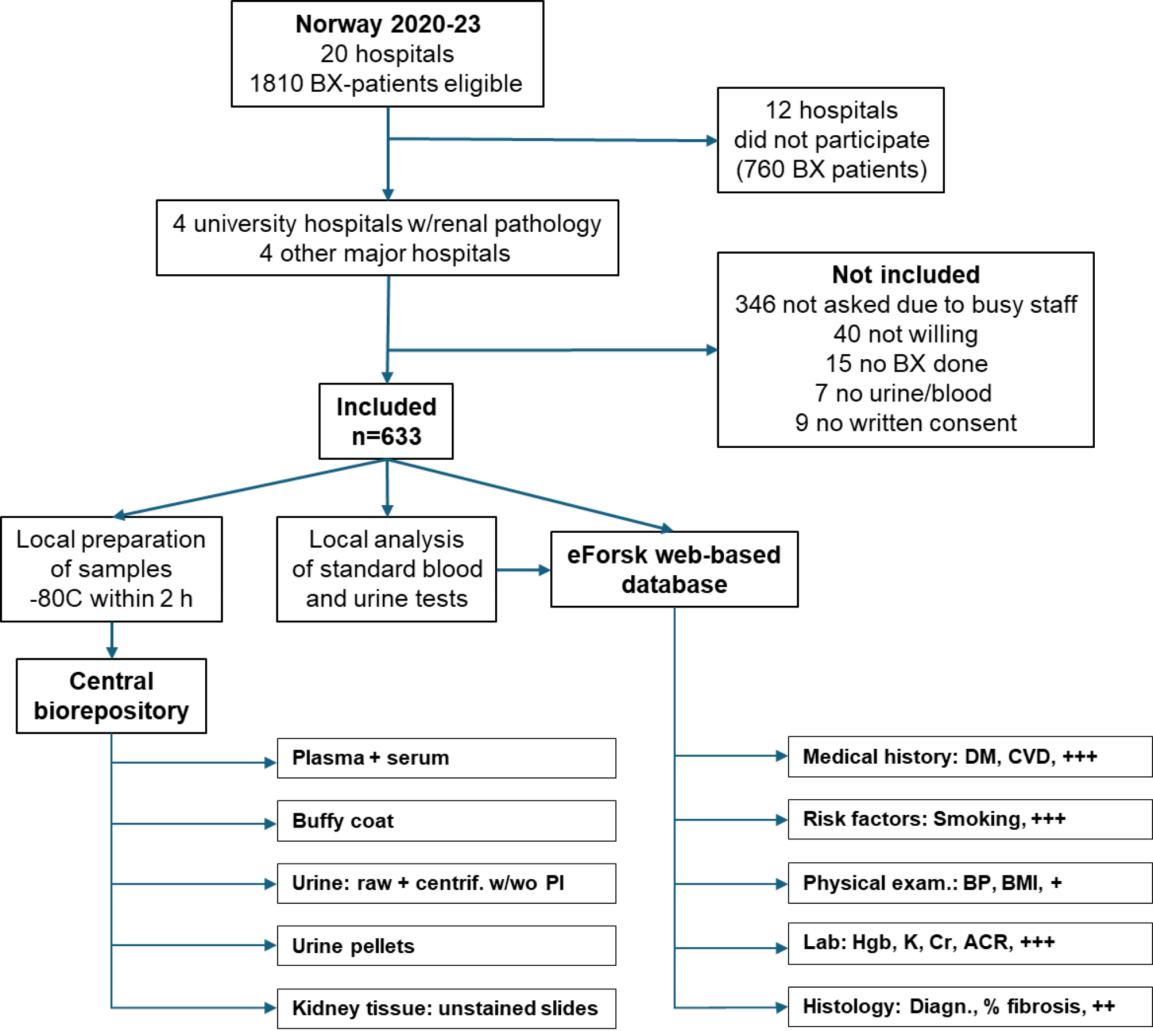
Flowchart displaying inclusion of patients, handling of data, and types of stored information and samples in the biobank. Note: we did not count as eligible participants the biopsy patients that were biopsied before the centers had formally started to recruit. Therefore, the number of eligible patients was 1810, a number slightly lower than the total number of 2395 biopsies in Norway for 2020-23

**Fig. 3:**
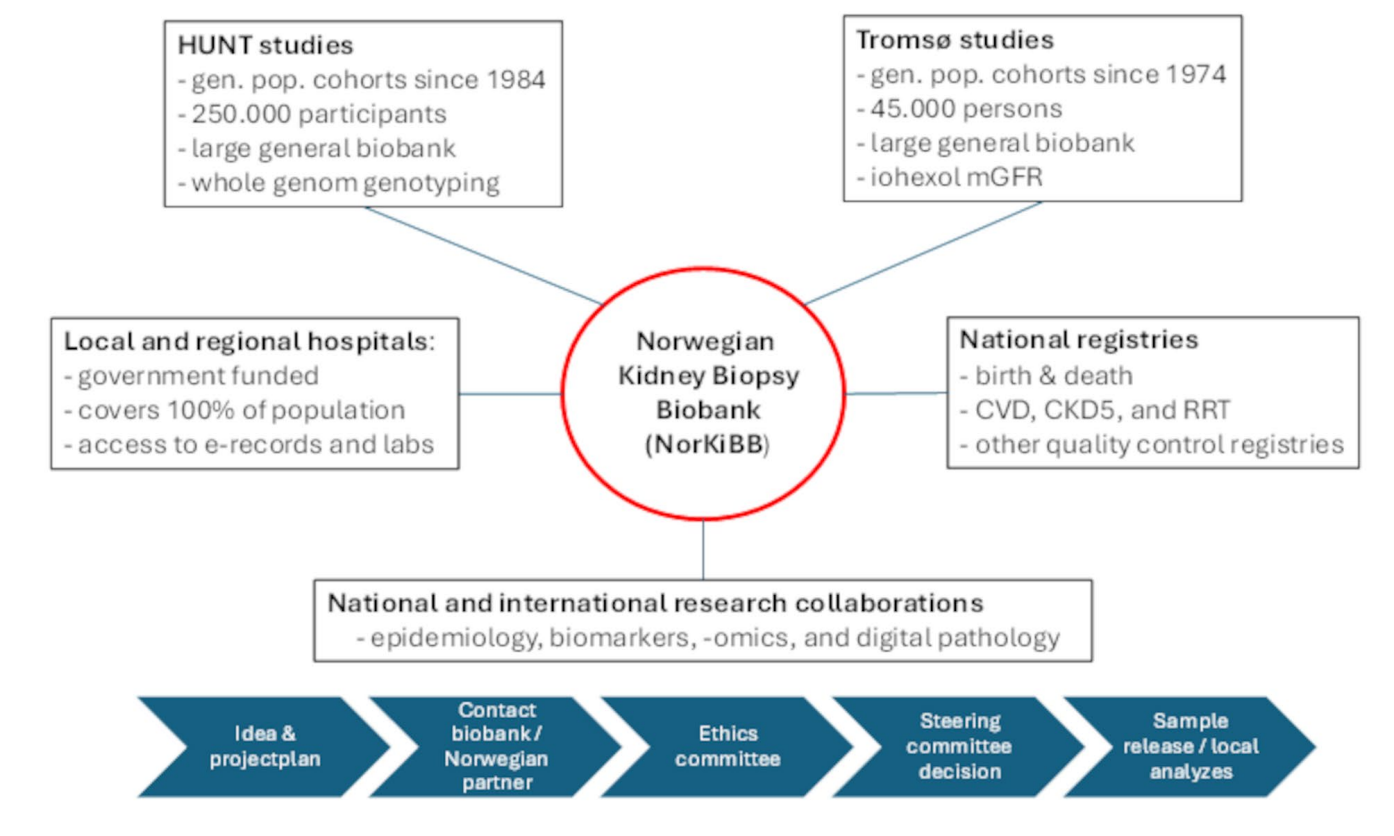
Organization of the biobank with main analytical platforms, outcome sources and collaborators

During the 3-year inclusion period, patients were included from all six university clinics in Norway (St. Olavs Hospital, Akershus University Hospital, Haukeland University Hospital, Oslo University Hospital Ullevål, Stavanger University Hospital, and the University Hospital of North Norway), see Fig. 1. In addition, two local hospitals (Levanger and Lillehammer) included a substantial number of patients, ensuring nationwide coverage. Figure 2 shows the inclusion process and gives an overview of the biological samples stored at BioBank1. Figure 3 shows the principles of information flow into the biobank from its collaborators and their organization.

A total of 633 patients (249 women) out of a total of 1050 patients with kidney biopsies were included, yielding a patient participation rate of 60%. The participants had a mean age of 52.6 years (SD 18.7), and hypertension, diabetes, and cardiovascular diseases (CVD) were highly prevalent at inclusion (83%, 16%, and 23%, respectively). Further details of medical history and risk factors by sex are given in Table 1. Most participants had moderately reduced kidney function (eGFR 30–59 mL/min/1.73m^2^, *n* = 206), but we also included many patients with normal or mildly reduced kidney function (eGFR > 60 mL/min/1.73m^2^, *n* = 206) and severely reduced kidney function (eGFR < 30 mL/min/1.73m^2^, *n* = 180). Mean eGFR was 53 ml/min/1.73m^2^, and mean albumin-to-creatinine ratio (ACR) was 164 mg/mmol. Further details on electrolytes, hematology, acid-base balance, vitamins, lipids, and hormones are provided in Table 2.

**Table 1:** Patient characteristics, risk factors and medical history at time of kidney biopsy

	Female	Male	Total
N	249	384	633
Age (years)	52.7 (18.4)	52.5 (18.9)	52.6 (18.7)
BMI (kg/m2)	26.5 (5.8)	28.1 (10.3)	27.4 (8.9)
General health (1, bad; 4, excellent)	2.7 (0.9)	2.7 (0.8)	2.7 (0.8)
Ever-smoking	104 (42%)	190 (49%)	294 (46%)
Hypertension	191 (77%)	334 (88%)	525 (83%)
Duration of HT (years)	7.2 (8.7)	8.0 (8.4)	7.7 (8.5)
Number of anti-hypertensive drugs	1.2 (1.2)	1.6 (1.4)	1.5 (1.3)
Systolic BP (mmHg)	129.9 (18.3)	135.3 (15.3)	133.2 (16.8)
Diastolic BP (mmHg)	76.8 (9.2)	79.6 (9.4)	78.5 (9.4)
ACEi/ARB (%)	107 (41%)	205 (52%)	312 (48%)
Diabetes (%)	26 (10%)	74 (19%)	100 (16%)
Diabetes duration (years)	13.7 (10.8)	15.6 (11.2)	15.1 (11.1)
DM-complications (%)	9 (35%)	41 (55%)	50 (50%)
CVD (%)	35 (14%)	108 (28%)	143 (23%)
Autoimmune diseases (%)	58 (22%)	79 (20%)	137 (21%)
Chronic infections (%)	5 (2%)	11 (3%)	16 (2%)
Chronic liver disease (%)	11 (4%)	5 (2%)	16 (2%)
Chronic obstructive lung disease (%)	22 (8%)	23 (6%)	45 (7%)
History of cancer (%)	33 (13%)	39 (10%)	72 (11%)

Note: Continuous variables are given as mean (1 standard deviation). Categorical variables are given as number of patients with the reported variable (percentage of all patients with info on the variable). General health is self-reported categories of bad (1), not so good, good, or excellent (4); hypertension is > 140/90 or treated; HT duration and number of anti-hypertensive drugs are among those with HT; DM duration and complications are among those with DM; DM-complications are physician-reported non-kidney complications like retinopathy, neuropathy, or amputations; CVD is self-reported myocardial infarction, angina pectoris, revascularization, or cerebral stroke; autoimmune diseases are self-reported SLE, ANCA vasculitis, Henoch Schonlein purpura, other vasculitides, other autoimmune disease; cancer does not include non-melanoma skin cancer

**Table 2:** Kidney function panel tests measured 0–3 days before the kidney biopsy procedure by eGFR range

	eGFR ≥ 90	eGFR 60–89	eGFR 30–59	eGFR 15–29	eGFR < 15	Total
N (%)	113 (19.1)	93 (15.7)	206 (34.8)	113 (19.1)	67 (11.3)	592 (100)
Hgb (g/dL)	13.5 (1.7)	13.6 (2.1)	12.7 (2.2)	11.2 (1.7)	9.9 (1.5)	12.4 (2.3)
K (mmol/L)	4.06 (0.40)	4.26 (0.71)	4.28 (0.51)	4.42 (0.62)	4.45 (0.68)	4.28 (0.6)
Phosphate (mmol/L)	1.10 (0.20)	1.06 (0.24)	1.12 (0.36)	1.26 (0.26)	1.90 (0.59)	1.22 (0.42)
Calcium - ionized (mmol/L)	1.24 (0.04)	1.22 (0.04)	1.24 (0.06)	1.22 (0.06)	1.14 (0.08)	1.22 (0.06)
PTH (pmol/L)	4.3 (5.4)	6.9 (4.6)	9.9 (9.3)	14.4 (9.9)	30.1 (17.8)	11.6 (12.1)
Creatinine (umol/L)	66.5 (13.3)	97.9. (17.9)	145.4 (30.7)	233.9 (53.3)	578.7 (261)	188.8 (176)
eGFR (mL/min/1.73m2)	111.6 (12.9)	72.7 (8.9)	43.3 (8.3)	23.5 (4.4)	8.8 (3.3)	53.2 (34.8)
Urea (mmol/L)	4.9 (1.7)	7.3 (3.3)	10.5 (7.1)	14.7 (5.8)	26.2 (11.8)	11.5 (8.9)
HCO3 (mmol/L)	26.5 (2.8)	26.5 (3.0)	24.9 (3.1)	23.6 (3.2)	21.9 (4.5)	24.8 (3.6)
Albumin (g/L)	38.1 (7.2)	35.4 (8.7)	39.6 (6.8)	38.3 (6.7)	33.3 (5.3)	37.7 (7.3)
25-OH-vitamin D (nmol/L)	50.6 (33.8)	47.8 (29.2)	59.5 (28.6)	68.1 (32.4)	47.6 (28.1)	56.2 (31.2)
u-dipstick hemoglobin (0–4)	1.8 (1.4)	2.0 (1.6)	1.5 (1.5)	1.4 (1.5)	2.7 (1.4)	1.7 (1.5)
u-ACR (mg/mmol)	150.7 (214)	241.0 (268)	130.1 (180)	147.6 (203)	207.4 (281)	163.9 (222)

Note: Values for each eGFR range are given as means (SD)

Indications for kidney biopsy are displayed in Table 3. Unexplained progressive CKD was the most common indication (288 of 592 participants, 45%), while 87 participants (14%) had nephrotic syndrome. There was no difference between men and women (as tested using the chi square test, data not reported), and the syndrome was equally common in patients above or below the age of 50 (12.3 vs. 14.4%, *p* = 0.28). Similar data are given for the other kidney biopsy indications and compared with data for all kidney biopsies performed in Norway in the four years of 2020-23.

**Table 3:** Indication for biopsy and histology verified diagnosis in patients included in the NorKiBB biobank by sex and age, and for all 20 kidney biopsy centers in Norway in total

	Sex		Age (years)		Total	
	Female	Male	< 50	≥ 50	NorKiBB (2020-23)	Norway (2020-23)
**Indications for biopsy (N)**	249	384	277	356	633	2395
Nephrotic syndrome	29 (12%)	58 (15%)	33 (12%)	54 (15%)	87 (14%)	438 (18%)
Nephritic syndrome	33 (13%)	49 (13%)	47 (17%)	35 (10%)	82 (13%)	337 (14%)
Acute kidney disease	53 (21%)	74 (19%)	36 13%)	91 (26%)	127 (20%)	704 (29%)
Chronic kidney disease	94 (38%)	194 (51%)	110 (40%)	163 (46%)	288 (45%)	796 (33%)
Isolated hematuria / Proteinuria	40 (16%)	9 (2%)	51 (18%)	13 (4%)	49 (8%)	120 (5%)
**Histologically verified diagnoses (N)**	234	358	255	337	592	2395
Minimal Change Disease	11 (5%)	13 (4%)	9 (4%)	15 (5%)	24 (4%)	114 (5%)
Focal Segmental Glomerulosclerosis	9 (4%)	16 (5%)	15 (6%)	10 (3%)	25 (4%)	83 (3%)
Membranous Glomerulonephritis	9 (4%)	17 (5%)	8 (3%)	18 (5%)	26 (4%)	98 (4%)
Membranoproliferative	4 (2%)	3 (1%)	2 (1%)	5 (2%)	7 (1%)	38 (2%)
Mesangioproliferative incl. IgA	46 (20%)	88 (25%)	94 (37%)	40 (12%)	134 (23%)	388 (16%)
ANCA vasculitis	15 (6%)	23 (6%)	9 (4%)	29 (9%)	38 (6%)	143 (6%)
Lupus nephritis	17 (7%)	8 (2%)	19 (8%)	6 (2%)	25 (4%)	76 (3%)
Other glomerulonephritis	5 (2%)	10 (3%)	3 (1%)	12 (4%)	15 (3%)	121 (5%)
Arterionephrosclerosis	27 (12%)	56 (16%)	18 (7%)	65 (19%)	83 (14%)	142 (6%)
Diabetic nephropathy	13 (6%)	42 (12%)	16 (6%)	39 (12%)	55 (9%)	160 (7%)
Tubulo-interstitial nephropathy	22 (9%)	32 (9%)	18 (7%)	36 (11%)	54 (9%)	176 (7%)
Myeloma / light-chain disease	12 (5%)	6 (2%)	7 (3%)	11 (3%)	18 (3%)	19 (1%)
Amyloidosis	7 (3%)	11 (3%)	1 (0%)	17 (5%)	13 (2%)	88 (4%)
Alport’s	4 (2%)	5 (1%)	7 (3%)	2 (1%)	9 (2%)	12 (1%)
Fabry’s	3 (1%)	3 (1%)	0 (0%)	6 (2%)	6 (1%)	18 (1%)
Acute tubular necrosis	5 (2%)	8 (2%)	5 (2%)	8 (2%)	13 (2%)	79 (3%)
Other non-GN kidney disease	18 (8%)	5 (1%)	13 (5%)	10 (3%)	28 (5%)	62 (3%)
Normal	7 (3%)	12 (3%)	11 (4%)	8 (2%)	19 (3%)	56 (2%)

IgA glomerulonephritis was the most common diagnosis (23%), while arterionephrosclerosis was the second most common diagnosis (14%). Similar findings were reported for the total of Norway. The distribution of the full range of histological diagnoses is displayed in Table 3. Indications and histology verified kidney diagnoses were very similar among those included in the NorKiBB over the 3-year period and for the total of Norway. Furthermore, country level data on kidney health and related measures of relevance for the generalizability to other European countries and the US are shown in Table 4.

**Table 4:** Comparison of CKD prevalence and population level measures of importance for kidney health in Norway, United Kingdom, and USA based on published data

	Norway	UK	USA
CKD prevalence (%)	11.1	12.8	13.5
National key numbers			
Age (median total population)	39.5	40.6	38.5
Life expectancy at birth (years)	82.9	80.1	76.4
Health expenditure per capita (USD)	9021	5738	12,012
Wealth inequality (Gini index)	27.7	32.4	39.8
Universal health coverage (%)	> 80	> 80	> 80
Modifiable CKD risk factors			
Overweight or obese (%)	52	64	68
Current smoking (%)	8.0	11.2	8.9
Diagnosed diabetes mellitus (%)	3.6	6.3	10.7
Hypertension (%)	30.5	26.4	31.6
End-Stage Renal Disease			
ESRD incidence per million inhabitants	99	108	383
eGFR at RRT start (ml/min/1.73m2)	9.0	7.0	9.5
Age at RRT start (years)	62.3	61.0	62.5
Transplanted (% of prevalent RRT)	67.2	56.4	30.4
Kidney biopsy incidence (pmp/y)	98	120	175
Kidney diagnosis causing ESRD (% of total)			
DKD	17	30	39
HN	32	8	27
GN	16	13	15
Cystic kidney disease	10	7	5
Other/unknown	25	42	14
Primary glomerulonephritis spectrum (%)			
Minimal change	14	18	11
FSGS	10	18	39
Mesangioproliferative incl. IgA	46	37	10
Membranous	12	21	13
Other GN	18	6	27

Note: Data are collected from published studies and the websites of well renowned international organizations like World Health Organization, Organization of Economic Cooperation and Development, Center of Disease Control, International Diabetes Federation, and national kidney registries. Information is from the most recent period available for all regions (primarily 2020-22), and more detailed information on references and websites are available in Supplementary Table 2

## Discussion

We demonstrate the feasibility of establishing a relatively large national kidney biobank across a variety of clinical and histopathologic diagnoses. During the first three years, blood and urine from 633 patients were stored, accompanied by kidney tissue, at a moderate cost. By providing high-quality biospecimens and deep clinical phenotyping, the NorKiBB aims to provide important new knowledge for patients with kidney disease.

### Brief comparison with other major kidney biobanks

Many large well-established biobanks like CRIC (US) [11], UK Biobank [12], BIND-NL (Netherlands) [13], CKD-REIN (France) [14], KORA (Germany) [15], and CKD-JAC (Japan) [16] contribute important information on CKD prevalence, risk factors, biomarkers, and clinical outcomes, but concurrently lack kidney biopsies that could provide research opportunities and accuracy. However, several well-established US biobanks with biopsy material of high quality have contributed to high impact publications [17–19], but the sample sizes used in prior manuscripts have been relatively modest and the disease spectrum has appeared different from other regions (e.g. higher prevalence of diabetes and FSGS). More recently, several new kidney biopsy biobanks have been established worldwide: NURTuRE-CKD (UK) [20], KORNERSTONE (Korea) [21], BMCKD (Canada) [22], CKD Biobank Australia [23], KPMP (USA) [24], and others. These have included relatively large numbers of patients, are well organized, and are often accompanied by very strong laboratory services. A potential strength of NorKiBB, compared to several of the mentioned biobanks, is the incorporation of deep phenotyping with extensive information on medical history, biochemistry measurements, and histology. This is increasingly recognized as important for modern research.

### Generalizability

Indications and histological diagnosis obtained on biopsy were quite similar in NorKiBB and across all biopsies conducted in Norway during the same time. There are, however, more arterionephrosclerosis patients in the NorKiBB biobank than in the national Norwegian registry. The difference is statistically significant when evaluated using the chi square test, at a p level of < 0,0001. One explanation for this may be that our study participants were more likely included and biopsied if they had a chronic kidney disease, rather than acute kidney disease. Including participants is more often accomplished in an elective and premeditated setting where there is time to plan ahead. In the acute setting, by contrast, it is easier to forget to fill in inclusion forms prior to the biopsy, or there is simply not enough time or people available to do the paper work before the biopsy. A minority of the acute biopsies are also carried out on weekends and in on-call settings outside of office hours, where staffing is lower. This may add to why we had much more chronic than acute diseases among our diagnoses. Of the chronic kidney diseases, arterionephrosclerosis is among the most frequent in most biopsy materials. We suppose that over time the relative frequency of arterionephrosclerosis diagnoses in our study will approximate the national frequency. All in all, we believe that studies based on our biobank will provide data representative for Norway, and the country level data on kidney health presented in the results section indicate that many results should be generalizable to other European countries and the US as well (see Table 4 and references in the Supplementary 3). All countries are high-income economies allocating large resources to healthcare. CKD prevalence is rather similar and mainly driven by life-style related risk factors like hypertension, diabetes, and overweight in each case. There are some notable differences. These lifestyle risk factors are somewhat less prevalent in Norway than in the US, rates of progression to ESKD are lower, and transplant availability is higher in Norway. The spectrum of primary glomerulonephritis is also somewhat different, with lower prevalence of FSGS and higher IgA nephropathy prevalence in Norway. However, whether this will influence the results from NorKiBB is uncertain. At minimum, the relatively large sample size of NorKiBB will allow evaluation within disease categories, for example among IgA nephropathy patients, and will allow comparison of consistency of relationships of risk factors and biomarkers with histopathological features across etiologies of CKD.

### Feasibility

There is a long list of important factors, beyond those related to the specific scientific topic, to consider when establishing a medical biobank: acceptability to participants, availability of financial, structural, and human resources, and a myriad of practical issues. Norway has a more than 100 years long tradition of medical registries [25], a strong data protection policy, and a population with high trust in political and governmental institutions. Participation rates in medical registries and studies have therefore been very high [26, 27]. Concordantly, 60% of eligible kidney biopsy patients were included in the NorKiBB. We did not obtain reasons for why the other candidate patients declined to participate, but local nephrologists have consistently reported the number of non-willing patients to be very few.

Furthermore, we demonstrate that biobanking from patients scheduled for kidney biopsy as part of their clinical management in a government funded health care system can be done with rather limited resources. In our study, physician visits, administrative contacts, blood and urine sampling, kidney biopsy with the radiologist, and a wide range of standardized blood, urine and histology analyses have all been done as part of routine clinical care which were leveraged for research purposes here. In addition, we used facilities and services from an already established biorepository (Biobank1), which was critical to enable us to start the NorKiBB with rather limited resources. Similar conditions may be present in other countries, and we strongly encourage the start-up of more kidney biobanks to improve highly needed research in the field, and the opportunity to compare and contrast results across populations.

### Limitations

The NorKiBB biobank has important limitations. Although inclusion rates have been high and we already have included a reasonable number of patients with a representative range of kidney diseases, the ability to study specific types of glomerulonephritis could be limited. For example, we have currently only enrolled 24 patients with minimal change disease and 38 patients with ANCA vasculitis. However, NorKiBB has excellent research possibilities for more common diseases like IgA nephropathy, interstitial nephritis, as well as for studying more general issues like tubular function and the longitudinal effects of crucial kidney risk factors like hypertension, diabetes, and obesity. Because we utilized kidney biopsies done for clinical indications, there is clinical selection of patients who may benefit from biopsy, which likely resulted in oversampling of glomerular diseases, but this reflects current medical practice. Most patients only have a kidney biopsy at a single time point, so it is not possible to study histological changes over time. The vast majority of patients are white, and generalizability to minority populations in the US and Asian populations can therefore not be assumed. The biobank has not collected information on dietary habits or fecal samples for microbiome examination, which are increasingly recognized as important for kidney research.

### Future projects

Biobanking has been crucial for developing personal or precision medicine, especially in oncology where we now see practical clinical benefits for a growing number of patients [28]. Similar progress should be possible in other fields, including nephrology. NorKiBB could be an important contributor in this work by combining a high-quality biobank with large high-quality general population-based cohorts, national quality registries, and a common, well-organized health care system that includes all Norwegian citizens. The biobank will be accessible for other research groups and can potentially contribute to many high-quality projects. Interested investigators from the study sites and collaborators can submit project proposals, and the steering committee will review the application for scientific quality, novelty, and suitability of the NorKiBB for the study question.

## Conclusions

Establishing high-quality kidney biopsy biobanks can be done with limited resources as part of patients’ standard care. Data from NorKiBB demonstrate that representative biobanks of sufficient size can be established within a rather short period of time and with limited financial resources. We encourage other kidney research groups and societies to establish their own biobanks and invite researchers to collaborate on future projects based on the rich material available in NorKiBB.

## Electronic supplementary material

Below is the link to the electronic supplementary material.

Supplementary Material 1

## Data Availability

Data is provided within the manuscript or supplementary information files.

## Abbreviations

ACR: Albumin-to-creatinine ratio
ANA: Antinuclear antibodies
ANCA: Antineutrophil cytoplasmic antibodies
BIND-NL: Biobank of Nephrological Diseases in the Netherlands
BMCKD: Biobank for the Molecular Classification of Kidney Disease
CKD: Chronic kidney disease
CKD-JAC: Chronic kidney disease–Japanese Cohort
CKD-REIN: Chronic kidney disease–Renal Epidemiology and Information Network
C3: Complement 3 protein
C4: Complement 4 protein
C1q: Complement 1q protein
CRIC: Chronic Renal Insufficiency Cohort
CVD: Cardiovascular disease
eGFR: Estimated glomerular filtration rate
EDTA: Ethylenediaminetetraacetic acid
ESKD: End stage kidney disease
GDPR: General Data Protection Regulation
HE: Hematoxylin-eosin
IgA: Immunoglobulin A
IgG: Immunoglobulin G
IgM: Immunoglobulin M
KORA: Cooperative Health Research in the Region of Augsburg
KORNERSTONE: KOrea Renal biobank NEtwoRk System TOward NExt-generation analysis
KPMP: Kidney Precision Medicine Project
NorKiBB: Norwegian Kidney Biopsy Biobank
NTNU: Norwegian University of Science and Technology
NURTuRE-CKD: National Unified Renal Translational Research Enterprise
PAS: Periodic acid-Schiff
RNA: Ribonucleic acid
RRT: Renal replacement therapy
SD: Standard deviation
SOP: Standard operating procedure
