# Supplementary material for: Norwegian kidney biopsy biobank (NorKiBB): organization, baseline characteristics, and generalizability of a low-cost national biobank
Source: BMC Nephrol. 2025 Feb 13;26:76. doi: 10.1186/s12882-025-04007-4 (PMC11827217; doi:10.1186/s12882-025-04007-4)
Supplement: Supplementary file 1 — Supplementary Material 1 [file 12882_2025_4007_MOESM1_ESM.docx]

**SUPPLEMENTARY MATERIAL**

**supplemenT 1.**

**List of all variables recorded At biopsy**

**General information:**

Center
Sex
Age
Biopsy date
Clinical suspected kidney diagnosis (free text)

**Clinical indication for biopsy:**

Nephrotic (Y/N)
Nephritic (Y/N)
Acute kidney failure (Y/N)
Chronic kidney disease (Y/N)
Isolated Proteinuria / Hematuria (Y/N)

**Biopsy information:**

Biopsy needle size (G)
Number of punctures
Biopsy operator (nephrologist / radiologist / other)
Complications (none / macroscopic hematuria / transfusion / intervention)
Outpatient (Y/N)

**Medical history:**General condition (Bad / not so good / good / excellent)
Hereditary kidney disease (Y/N)

Autoimmune disease (Y/N)
Autoimmune disease (free text: type year))

DM (Diabetes Mellitus) (Y/N)
DM type 2 (Y/N)
DM duration (years)
DM complications (Retinopathy / Nephropathy / Autonomic dysfunction / Amputation / Neuropathy)

Hypertension (Y/N)
Hypertension (free text: duration, complications)
Hypertension (number of BP medications)
ACE inhibitor (Y/N)

Cancer (Y/N)
Cancer (free text: organ year)
Chronic infection (Y/N)
Chronic infection (free text: type year)
Liver disease (Y/N)
COPD (Y/N)
COPD (grade 1-4)

Cardiovascular disease:
Myocardial infarction (Y/N)
Myocardial infarction (free text: year)
Angina or PCI (Y/N)
Angina or PCI (free text: year)
Stroke (Y/N)
Stroke (free text: year)
Other CVD (Y/N)
Other CVD (free text: year)
Heart failure (Y/N)
Heart failure NYHA class (1-4)

Smoking (never / former / current)
Exercise (Y/N)
Exercise intensity (low / medium / high)
Exercise duration (h / week)

**Medication**
All regular medication (free text: type of drug (name) – dose – frequency)
Nephrotoxic medications recently (NSAIDS, aminoglycosides, lithium, others)

**Medical examination:**Height
Weight
Systolic blood pressure
Diastolic blood pressure
Hear rate

**Standard kidney panel of blood and urine tests:**

Hemoglobin
White blood cells (LPK)
Platelets (TPK)
C-reactive protein (CRP)
INR
APTT
Sodium (Na)
Potassium (K)
Chloride (Cl)
Calcium (Ca)
Ionized calcium (iCa)
Phosphate (P)
Creatinine
eGFR
Carbamide (urea)
Bicarbonate (HCO3)
Albumin
Uric acid (Urat)
Parathyroid hormone (PTH)
Vitamin D
Glucose
HbA1c
Cholesterol
HDL
LDL
Triglycerides
Urinary sodium (uNa)
Urinary potassium (uK)
Urinary chloride (uCl)
Urinary creatinine (uKreat)
Urinary ACR
Urine pH (upH)
Urine erythrocytes (uEry)
Urine albumin (uAlb)

**Serological testing:**Monoclonal component in serum (type, concentration)
Monoclonal component in urine (type, concentration)
ANA
Anti-GBM
PR3
MPO
Cryoglobulins
PLA2R

**Histology report:**

Primary diagnosis (free text)
Primary diagnosis (ICD-10 code)
Secondary diagnosis (free text)
Secondary diagnosis (ICD-10 cose)
GN group
Non-GN group
Number of glomeruli
EM (electron microscopy) (Y/N)
Chronicity index
Glomerulosclerosis (0-3)
Tubular atrophy (0-3)
Interstitial fibrosis (0-3)
Vascular sclerosis (0-3)
Total sum chronicity index (0-10)
Primary report (full report as free text)
Secondary report (revised report by study pathologists)

**Supplement 2**

**Patient information paper**

**NTNU, 1. June 2020**

**REQUEST TO DONATE BIOLOGICAL MATERIAL FOR KIDNEY MEDICINE RESEARCH**

**BACKGROUND AND PURPOSE**

The kidney medicine research group at St. Olav Hospital / Norwegian University of Science and Technology (NTNU) in Trondheim aims to acquire new medical knowledge and determine how the treatment of each individual patient can be improved. All Norwegian patients undergoing a kidney biopsy are now being asked to donate urine and blood to develop better diagnostic tools and treatments. This work is supported by the Norwegian Kidney Registry, a national quality registry organized by Norwegian nephrologists, and is funded by the Norwegian Research Council.

**WHAT BIOLOGICAL MATERIAL WILL BE COLLECTED?**

Urine, blood, and kidney tissue will be collected. This is done as part of the regular examinations you are currently undergoing. No additional samples or needle sticks will be performed, and no extra tests or examinations will be conducted in the future:

- **Urine sample**: We will collect approximately 100 ml of extra urine.
- **Blood sample**: No extra needle sticks, but we will collect about 30 ml of extra blood.
- **Kidney tissue**: No extra needle sticks; we will only store the leftover kidney tissue from the regular examination.

The collection will not involve any additional procedures, discomfort, or risk beyond what your regular kidney doctor has already ordered. Blood and urine samples will be stored at Biobank1 (Health Mid, Trondheim), while kidney tissue will be stored locally.

**BROAD CONSENT**

By donating biological material to this general research biobank, you also give broad consent for the material and relevant health information to be used in future research related to kidney diseases.

**COLLECTION AND USE OF HEALTH INFORMATION**

The biobank will contain some information about you, such as your name, personal identification number, diagnosis, and treatment facility. These details are only accessible through a linkage key designed to protect your identity. The institution is responsible for safeguarding and managing the linkage key. Biobank1 has extensive experience and solid procedures to ensure the security of your information.
The material and information about you will be stored permanently and may be used in specific research projects. Researchers will never have access to your name, personal identification number, or other identifying information.

**GENETIC STUDIES**

No genetic studies will be conducted to provide information about your hereditary traits concerning the risk of other future diseases (diabetes, heart disease, Alzheimer's, etc.).
However, since all processes in kidney cells are controlled by our genetic material, various gene-based techniques will be used to study your kidney disease. This may involve examining the tissue sample to determine the quantity or location of certain proteins or gene products. It may also include analyzing the genetic risk for kidney diseases not following a typical inheritance pattern but where certain gene variants seem to predispose for kidney disease to a minor or moderate extent. This information is of little significance to you as an individual (and you will therefore not receive the results of your sample), but it will be essential for understanding why kidney disease occurs, how it develops, and how it can be treated.

**INFORMATION ON FUTURE PROJECTS**

All future research projects using material from you must be pre-approved by a regional committee for medical and health research ethics, but you will only rarely be asked again for permission to use your material. In some research projects, it may be relevant to combine information from the biobank with details from medical records, health surveys, health registries, or public administrative registries. It may also be relevant for biological material to be shared with research institutions in Norway and abroad for analyses that we do not have the capacity for. The material will be shared without your name, personal identification number, or other direct identifying information. Information about future research projects will be made available on CRISTIN (information system for all ongoing research in Norway, see [www.cristin.no](http://www.cristin.no)).

**PARTICIPATION IS VOLUNTARY**

Donating biological material is voluntary and requires your consent. It will not affect your treatment if you choose not to donate a sample, or if you later wish to withdraw your participation.

**ABILITY TO WITHDRAW CONSENT, ACCESS, MODIFY, AND DELETE INFORMATION**

You can request access to the material stored from you at any time. You may also request that the material be destroyed without needing to provide a reason. However, the destruction of the material will not result in the deletion of derived information that has been included in compilations or analyses.

**CONTACT**

Responsible for the biobank: NTNU / Central Norway Health Trust
Contact person: Professor / Senior Consultant Stein Hallan (switchboard: 7257 3000).

**CONSENT TO STORE BIOLOGICAL MATERIAL**

I am willing to give broad consent for my biological material to be stored permanently and used in future research.

Place and date: ____________________ Participant's signature: ____________________

Participant's name in printed letters: ____________________

**SUPPLEMENT 3**

**References and links to information given in Table 4:**

1. **Kidney biopsy incidence:**
   https://nephro.no/nnr/AARSRAPPORT_NNR_2022.pdf
   <https://karger.com/ajn/article/43/1/1/326192/Renal-Biopsy-in-2015-From-Epidemiology-to-Evidence>
   <https://www.srr.scot.nhs.uk/Projects/PDF/SSR-Report-2017/Appendix-N-2018-10-09-SRR-Report.pdf>
2. **CKD prevalence**:
   <https://nccd.cdc.gov/ckd/detail.aspx?Qnum=Q372>
   <https://www.sciencedirect.com/science/article/pii/S0085253816301703>
   <https://www.kidneyresearchuk.org/wp-content/uploads/2023/06/Economics-of-Kidney-Disease-full-report_accessible.pdf>
3. **National key numbers:**
   <https://data.who.int/countries/578>
   <https://data.worldbank.org/indicator/SI.POV.GINI>
   <https://apps.who.int/nha/database/country_profile/Index/en>
   <https://www.who.int/teams/health-systems-governance-and-financing/global-monitoring-report-2021>
4. **ESRD:**
   <https://ukkidney.org/sites/renal.org/files/25th%20Annual%20Report%20Final%202.6.23.pdf>
   <https://nephro.no/nnr/AARSRAPPORT_NNR_2021.pdf>
   <https://usrds-adr.niddk.nih.gov/2022/reference-tables>
5. **CKD modifiable risk factors:**<https://stats.oecd.org/Index.aspx?DataSetCode=HEALTH_STAT>).
   <https://stats.oecd.org/Index.aspx?DataSetCode=HEALTH_STAT>.
   <https://data.who.int/countries/578>).
   <https://www.statista.com/statistics/236764/prevalence-of-diabetes-in-selected-countries/>

<https://diabetesatlas.org/data/en/country/148/no.html>

**Causes of ESRD**:
<https://usrds-adr.niddk.nih.gov/2020/reference-tables>
<https://ukkidney.org/sites/renal.org/files/UKKA%2026th%20Annual%20Report%20Chapter%202%20-%20Incidence%20-%202024-07-07.pdf>
<https://nephro.no/nnr/AARSRAPPORT_NNR_2022.pdf>

**Spectrum of GN**:
<https://www.ajkd.org/action/showPdf?pii=S0272-6386%2816%2930021-X>
<https://www.srr.scot.nhs.uk/Projects/PDF/SSR-Report-2017/Appendix-N-2018-10-09-SRR-Report.pdf>
<https://nephro.no/nnr/AARSRAPPORT_NNR_2022.pdf>
